# Homœopathic Surgery

**Published:** 1894-09

**Authors:** Wm. Keaney

**Affiliations:** De Soto, Mo.


### HOMŒOPATHIC SURGERY.

Wm. Keaney, M. D., De Soto, Mo.

February 20th, 1891.—Mrs. T., æt. forty-four years. Has suffered for the past eighteen months from a *fissura ani*.

Stooling is painful, burning pain, which keeps up after stool is over. Fæces, yellowish lumps covered with streaks of blood ; bleeding continues slightly for a little while after stooling; red sand in urine.

Has a good appetite, but a very little satisfies, and bloats so she has to loosen clothing after eating. Sour, watery eructations.

First one and then the other foot is cold.

Burning pain was so excruciating dreaded to stool, and suffered for two hours afterward from the pain. ℞. Lyc.$^{cm}$ (Swan) one dose.

March 4th.—Better in all respects for a week; an increase of symptoms for past two or three days. Lyc.$^{cm}$, one dose (Swan).

March 13th.—Better; little pain. Sac-lac.

From now on until April 20th kept her alternately using Sac-lac. powders and unsophisticated pellets, at which date she was discharged cured, and she has so remained to this day. As to her being cured there is no question; as to the diagnosis a couple of my predecessors (old school) diagnosed the same, *i. e.*, fissure of anus. No matter who may question the diagnosis, I removed all her symptoms, as I have in a case which I diagnosed as Hodgson's disease, curing it with two doses of Kali-carb.$^{dmm}$ (Swan). In this latter case I may have erred in the identification of the disease, but the patient is alive and well. *Cui bono*, if you make an unerring diagnosis, and the disease carries off the patient?

