# Correction in September Number

**Published:** 1894-10

**Authors:** Wm. Keaney

**Affiliations:** De Soto, Mo.


### CORRECTION IN SEPTEMBER NUMBER.

DE SOTO, MO., Sept. 23d, 1894.

EDITOR OF THE HOMŒOPATHIC PHYSICIAN:—Please take notice that in the publication of my case, on page 274, line 21, I am made to say that I had cured a case of Hodgson's disease with one dose of Kali-c. It should have been *Hodgkin's disease,* a glandular not an aortic trouble. Hope you will make the correction.

Yours fraternally,

WM. KEANEY.

